# Exploring nurse- and allied health professional-led opportunistic atrial fibrillation screening with artificial intelligence-enabled devices in community and primary care

**DOI:** 10.3389/fcvm.2026.1831475

**Published:** 2026-07-07

**Authors:** Lai Yin Leung, Lisa Pau Le Low

**Affiliations:** S.K. Yee School of Health Sciences, Saint Francis University, Hong Kong SAR, China

**Keywords:** allied health, artificial intelligence, atrial fibrillation, mass screening, nursing, primary care, wearable electronic devices

## Abstract

Undiagnosed atrial fibrillation (AF) is a leading cause of preventable ischemic stroke, particularly in the ageing populations. While traditional screening relies on physician-interpreted 12-lead electrocardiography (ECG), which is considered the gold standard, the increasing availiability of new artificial intelligence (AI)-enabled devices, such as single-lead ECG and photoplethysmography (PPG) tools, offer decentralised and scalable alternatives. This mini review argues that AI-enabled, nurse- and allied health professional (AHP)-led opportunistic screening represents a necessary paradigm shift in AF detection in the community and primary care setting. It is transitioning from a reactive, physician-dependent model to a proactive, community-based approach to provide early, timely and accessible interventions to reduce the risks of stroke. These devices have demonstrated good diagnostic accuracy when compared with ECG. Nurse-led opportunistic screening was found to be cost-effective and to reduce stroke risk by facilitating earlier anticoagulant initiation. Widespread adoption has been hindered by substantial barriers such as false positive results, a lack of standardised training, and liability concerns regarding AI interpretation. However, nurses and AHPs are uniquely positioned to lead opportunistic AF screening initiatives using AI technology. To maximise the clinical impact and solidify this new paradigm, future implementation strategies should prioritise workforce training, robust data governance, and the integration of AI findings into established clinical pathways to enable physician confirmation.

## 1 Introduction

Undiagnosed atrial fibrillation (AF), the most common type of cardiac arrhythmia, is a major public health concern due to its associations with increased risks of ischemic stroke and other complications. AF is known to increase the risks of clot formation and subsequent thromboembolic events, including a five-fold increased risk of ischemic strokes, with statistics indicating a preceding or concurrent AF diagnosis in 24% of ischemic stroke cases (1–3). It is also associated with a twofold increase in all-cause mortality risk (4). In 2021, the number of individuals affected by AF reached approximately 52.5 million worldwide (5), and the global age-standardised prevalence rate (ASPR) was approximately 620.5 per 100,000 population in 2021 (6).

According to the literature, asymptomatic AF is often discovered only after a stroke or other serious event has occurred, suggesting that an alarmingly large proportion of the population may be undiagnosed. In one Hong Kong study, 0.8% of the participants were newly diagnosed with AF, of whom 65.3% were asymptomatic (7). Another study of patients with ischemic stroke found that 9.4% had undiagnosed AF; among them, 21.1% were newly diagnosed with stroke, highlighting the tendency for AF to be newly identified only when a stroke occurs (8). Therefore, early detection is paramount for initiating appropriate and timely management of AF, including anticoagulation therapy to reduce the risk of stroke, and for facilitating screening efforts in community and primary care settings to prevent stroke (9).

To evaluate the efficacy of these detection methods, it is crucial to explicitly distinguish between the two primary screening strategies: systematic screening and opportunistic screening. Systematic screening involves actively inviting an entire targeted demographic (e.g., all individuals over age 65 in a region) for testing, regardless of their current health-seeking behaviour (9). In contrast, opportunistic screening leverages existing interactions with the healthcare system, testing patients when they present for routine check-ups, vaccinations, or chronic disease management (9). While mass screening provides comprehensive population coverage to discover undiagnosed AF, it is highly resource-intensive and logistically complex (4). This review focuses specifically on opportunistic screening, which requires fewer structural resources and aligns more closely with the routine clinical workflows of nurses and AHPs. To achieve this, a fundamental change is to reframe their roles beyond operational tasks and clinical workflows are required. This manuscript asserts that nurses and AHPs should be empowered to conduct opportunistic AF screening using AI-enabled devices and to decentralize detection away from the exclusively physician-directed diagnostics. This model offers a scalable, cost-effective, and highly accurate strategy to proactively combat the growing public health burden of undiagnosed AF.

## 2 Literature review

### 2.1 Developments in nurse and allied health professional (AHP)-led AF screening with AI-enabled devices

The driving this paradigm shift in AI-enenabled AF screening requires leveraging on the existing strengths of the primary care workforce. Indeed, Nurses and AHPs are already well-positioned to conduct opportunistic AF screening and detection in primary care and community settings, given their frequent face-to-face contact with high-risk patients who are present to the clinic during routine check-ups and chronic disease management, as well as through vaccination and health education programmes (9, 10). The European Society of Cardiology guidelines now include the implementation of opportunistic screening for all individuals aged 65 years or older who engage with healthcare systems (11). These guidelines also explicitly address the crucial roles of nurses and AHPs in screening, stroke risk assessment, and patient education as part of integrated AF management (11).

Pulse palpation by nurses, a standard and valuable method of initial AF screening in clinical settings, particularly primary care settings, has some limitations. For example, its reported sensitivity and specificity for detecting AF have been inconsistent across studies. One systematic review found that pulse palpation for AF detection had a pooled sensitivity of 94% [95% confidence interval (CI), 84%–97%] and a pooled specificity of 72% (95% CI, 69%–75%) (12). Other studies have reported sensitivity rates of 62.8%–79% due to variations in nurses' experience levels and uncertainty about pulse regularity or an inability to conduct palpation (13, 14). These studies have highlighted the need for supplementary diagnostic methods, such as electrocardiography (ECG), to confirm a diagnosis of AF. Studies have suggested that nurse-led pulse palpation and 12-lead ECG screening are more cost-effective compared with either no screening or AF screening using traditional methods (15). Currently, physician-directed 12-lead ECG remains the gold standard for AF screening and diagnosis (11). The accuracy of this approach varies, with one article reporting a sensitivity of around 80% and a specificity range of 85%–92% (16). ECG is also time-consuming for physicians and has substantial cost implications (17, 18).

Technological advancements in AF detection devices, especially artificial intelligence (AI)-enabled devices, offer promising alternatives that could replace or augment traditional pulse palpation methods. Nurses and AHPs must remain at the forefront of these developments and ensure that they are highly competent in performing and interpreting ECGs to identify AF patterns. This approach was successfully implemented in the SAFE study, in which practice nurses received education on AF detection (19). Rather than merely acting as operators of new technologies, nurses and AHPs must be recognized as clinical leaders who drive the entire screening continuum. This development has created ample opportunities for healthcare collaborations in which nurses and APHs are taking the lead in early AF screening initiatives by using AI-enabled devices to coordinate comprehensive patient care (11). Their expanded strategic and clinical roles have included:

- Providing clinical leadership by integrating AI screening protocols into established chronic disease management and preventive care pathways.
- Ensuring accurate data collection and performing initial clinical triage by evaluating AI-generated risk outputs in the context of the patient's holistic health profile.
- Driving shared clinical decision-making by stratifying patient risk and determining the clinical urgency for physician escalation.
- Acting as the critical bridge to confirm AF from AI-enabled device by performing a 12 lead ECG.
- Serving as central care coordinators in navigating patients through the complex transition from community screening to primary care and specialist cardiology services.
- Leading patient engagement and education by counselling individuals on their AI results, explaining the necessity of medical interventions (such as anticoagulation therapy), and empowering them to adopt targeted lifestyle modifications.

Through the collaborative partnerships, nurses and AHPs have become care navigators, who can autonomously assess risk, help to adjust patients' care plans as needed and facilitate proactive management to prevent stroke and related serious complications. This approach, when coupled with the use of AI-enabled devices to enhance early detection, can streamline the physicians' workflows, and allow them time to focus on the most complex, crucial aspects of stroke management and advanced interventions.

### 2.2 Integrating AI technology with AF screening

AI is emerging as a transformative force in healthcare. AI-enabled devices have demonstrated good accuracy and efficiency when detecting AF, particularly in asymptomatic individuals. These potentially wearable devices can be equipped with AI algorithms to accurately detect AF episodes and thus may be deployed widely for the early detection of arrhythmic episodes (20). Convolutional neural networks also have been used to precisely detect cardiac arrhythmias, including AF from noisy ECG signals (21, 22). AI has also been validated for use in cross-platform detection. One study demonstrated that a deep neural network (DNN)-based algorithm could detect AF across multiple smart devices, including smartwatches and handheld ECG devices, at levels of accuracy comparable to cardiologists' interpretation of 12-lead ECG (23).

As mentioned previously, ECG analysis remains the gold standard for accurate detection of AF episodes. Simple and convenient single-lead ECG devices can be incorporated into wearables, enabling the capture of ECG patterns for interpretation (24). A systematic review and meta-analysis of the accuracy of AI-based single-lead ECG for AF diagnosis reported a sensitivity of 92.3% (95% CI 88.9%–94.8%) and a specificity of 96.2% (95% CI 94.6%–97.4%). This sensitivity and specificity remained high even in outpatient settings, with respective rates of 90.7% (95% CI 76.8%–96.6%) and 98.1% (95% CI 95.1%–99.3%), and were maintained using more accessible AI devices rather than the 12-lead ECG screening (25).

Photoplethysmography (PPG) is another standard method of AF detection. This technique involves illuminating the skin with a light-emitting diode, then using a photodetector to detect the reflected light. The PPG signal represents a pulse pressure waveform, thus enabling heart rhythm monitoring. This signal can be used to detect R-R interval variability and AF using a specific algorithm (26). A 2024 study reported that an AI-enabled PPG device could detect AF within 5 s at an accuracy rate of 99.2% (27). A recent systematic review and meta-analysis of AI-enabled PPG for AF diagnosis reported a sensitivity of 95.1% (95% CI 92.5%–96.8%) and specificity of 96.2% (95% CI 94.3%–97.5%) (25). These findings demonstrate the promise of PPG based on deep learning techniques as a technological advancement for population-based arrhythmia screening (28).

Blood pressure monitors with AF detection algorithms are also available for identifying AF episodes. These devices incorporate machine learning techniques and are designed to detect irregular heart rhythms during blood pressure measurements. Their accuracy varies depending on the specific device and algorithm. A systematic review and meta-analysis of various blood pressure monitors with incorporated AF detection algorithms reported a pooled sensitivity of 97% (95% CI, 92%–99%) and specificity of 93% (95% CI, 90%–95%) (29).

Collectively, these robust diagnostic metrics across multiple AI-enabled modalities (single-lead ECG, PPG, and modified blood pressure monitors) represent more than just technological milestones; they provide the necessary clinical synthesis to justify a nurse- and AHP-led screening model. The consistently high sensitivity reported across these devices (generally exceeding 90%) ensures a low rate of false negatives, which is the paramount goal of opportunistic screening to prevent strokes. Simultaneously, the high specificity (often >95%) is equally critical for the viability of this decentralized model. By minimizing false positive results, these highly specific AI tools prevent the over-referral of healthy individuals to secondary care. Early detection has definite advantages, including a reduced time to diagnose and improved detection of clinically relevant arrhythmias (25). Nurses and APHs can take the lead in using AI-enabled devices to conduct AF screening programmes in larger populations. This precision is essential for protecting physician bandwidth, avoiding the inflation of downstream healthcare costs, and preventing unnecessary psychological distress for patients. Ultimately, these metrics validate that AI-enabled tools can reliably function as an autonomous triage layer in the community by effectively filtering high-risk patients, and reserving the gold-standard, physician-interpreted 12-lead ECG strictly for confirm the final diagnosis (30, 31).

### 2.3 Clinical outcomes and cost-effectiveness of AI-enabled devices

Study on the use of AI-enabled ECG devices have shown improvements in clinical outcomes in terms of better management and reduced complications and costs. The REVEAL AF study reported that 76% of undiagnosed AF patients had received interventions following AF detection, including the initiation of oral anticoagulation therapy in 63% of patients with detected AF, as well as rhythm or rate control pharmacotherapy, cardioversion, ablation, and cardiac specialist referral (30).

Incorporating nurses and APHs into AI-enabled AF screening programmes for high-risk populations has considerable implications for cost-effectiveness. Studies have shown that both systematic and opportunistic AF screening are cost-effective when compared with no screening or traditional AF screening methods (15). While large-scale trials like the STROKESTOP study have demonstrated that systematic, population-based mass screening for AF leads to significant reductions in stroke incidence and healthcare costs at a macro level, implementing such programmes require immense centralized logistical and financial resources (18).

Conversely, opportunistic screening has been highlighted as an optimal and more scalable approach, due to its cost-effectiveness and feasibility in primary care settings (31). The STROKESTOP study demonstrated that older adult population-based screening for AF is cost-effective, as it leads to significant reductions in stroke incidence and healthcare costs (18). Therefore, the integration of AI-enabled devices into opportunistic screening programmes may be a cost-effective and modern approach to AF detection that can be seamlessly implemented by nurses and APHs during existing patient encounters (15, 32).

The implementation of opportunistic screening was also found to be cost-effective in settings such as pharmacies and general outpatient clinics (33, 34). In high-risk populations, long-term continuous screening proved to be effective for detecting AF (35). AF screening was associated with significant reductions in the number of combined events pertaining to the primary endpoint of ischemic or haemorrhagic stroke, systemic embolism, bleeding leading to hospitalisation, and all-cause mortality [hazard ratio: 0.96 (95% CI 0.92–1.00); *p* = 0.045] (36).

AI-enabled screening tools have been associated with reductions in healthcare utilisation, in-person visits, and hospitalisations, all of which have led to cost savings (37, 38). A cost-effectiveness analysis conducted in rural Taiwan in 2023 compared AI-enabled 12-lead ECG screening with physician-directed screening. The authors found that AI-ECG screening was associated with lower costs and similar effectiveness, making it a practical alternative to traditional AF screening in areas with limited medical resources (39). Similarly, wearable AI-enabled devices have been shown to reduce the need for prolonged in-hospital monitoring, indicating the potential to reduce healthcare costs. This was supported by a study that evaluated wearable PPE and ECG devices and found that they were cost-effective when compared with either no screening or traditional AF screening methods (15).

## 3 Discussion

Although our review supports the use of AI in AF screening and diagnosis, realising this paradigm shift depends on overcoming several multifaceted barriers. To provide a structured and actionable analysis, we have categorised these challenges into technical, legal/ethical, clinical, and organisational domains, aligning each with targeted strategic solutions.

### 3.1 Technical barriers and solutions

First, AI models for AF detection are technically limited by poor generalisability and the inherent risk of algorithmic bias. Many AI algorithms are trained on retrospective datasets that often lack sufficient diversity and disproportionately represent specific demographic or geographic groups. Consequently, their accuracy can vary significantly across different populations, potentially leading to lower detection rates or higher false-positive rates in underrepresented demographic groups. Furthermore, the “black box” nature of many advanced AI algorithms presents a significant barrier regarding interpretability. While deep learning networks (such as those used in advanced ECG and PPG analysis) are highly accurate, they often do not provide a transparent rationale for their diagnostic outputs. This lack of interpretability makes it difficult for clinicians, including nurses and AHPs, to cross-check the AI's reasoning or explain the findings to the patient. AF detection also heavily depends on the quality and availability of data, and false positive results remain challenging (25).

Indeed, addressing this variability is paramount; therefore, standardised and robust external validation methods across diverse, real-world cohorts will be crucial. Future prospective trials must intentionally secure equitable representation across race, ethnicity, genders, and clinical comorbidities. Only by ensuring these algorithms perform accurately across all demographics can we guarantee their equitable applicability, ensuring that a nurse and AHPs in a low-resource rural clinic can trust the AI's diagnostic output as a specialist in a tertiary hospital. Integrating AI with multimodal data, including clinical and imaging data, could enhance risk stratification and personalised care management. Efforts should be made to standardise datasets to ensure diverse representation and improve data quality to ensure the generalisability and reliability of AI models. Additionally, future AI development must prioritize “explainable AI” (XAI) frameworks to provide clinicians with interpretable visualisations of the data points and algorithm's conclusions (40, 41).

### 3.2 Legal and ethical barriers and solutions

Second, compounded by this lack of transparency, questions remain regarding whether nurses and APHs are liable for errors or adverse outcomes associated with AI-driven decisions, such as a the failure of device to detect AF or tendency to produce false positive results. Nurses and APHs may therefore be hesitant to adopt AI-enabled devices (42). Addressing this hesitancy requires the immediate development of robust ethical and regulatory frameworks. These frameworks, co-designed by healthcare providers, legal experts, and AI developers, must explicitly define accountability and shared liability models when AI tools are used in healthcare to ensure that professionals are legally protected when following established AI-assisted clinical pathways. Robust ethical and regulatory frameworks are needed to address privacy, accountability, and equity concerns and should be developed collaboratively by healthcare providers, patients, and AI developers.

### 3.3 Clinical barriers and solutions

On a clinical level, false positives can cause patients to experience anxiety and unnecessary investigations. Nurses and AHPs may perceive AI as a threat to their professional autonomy or express scepticism about its benefits, leading to resistance and concerns about the effects of AI on their roles. To mitigate these clinical barriers, comprehensive training programmes that focus on the benefits and limitations of AI-enabled devices for nurses and APHs are needed. This type of education can reduce these professionals' resistance to change and improve the effective use of AI-enabled devices in primary and community settings. Nurses and APHs must educate patients on the limitations of these technologies and provide guidance regarding appropriate follow-up care. Furthermore, fostering direct collaboration between clinicians and AI developers during the design phase will ensure these devices are viewed as tools that empower, rather than create threat and loss of autonomy. For patients, proactive participation may improve the AF screening process, and increases thier autonomy, compliance, and beneficence (43).

### 3.4 Organisational barriers and solutions

At the organisational level, the successful integration of AI-enabled devices relies heavily on adequate resource allocation, particularly regarding time and training. Insufficient protected time for training is a substantial barrier in AF screening programmes (44–46), often leading to the suboptimal use of these technologies and fostering workforce resistance (47, 48). Compounding these logistical constraints are professional apprehensions: with some nurses expressing scepticism about the clinical benefits of AI or perceiving it as a direct threat to their professional autonomy, and further hindering adoption (49). Overcoming these challenges require a shift from top-down implementation to collaborative co-design. By actively involving frontline clinicians alongside IT specialists and AI developers during the design and validation phases, organisations can ensure these devices directly address real-world clinical needs and integrate seamlessly into existing workflows (45, 47). This collaborative approach not only resolves practical implementation challenges but also empowers nurses and AHPs, to reframe AI as a supportive clinical asset rather than an operational threat.

### 3.5 Global health perspective

Beyond optimizing workflows in well-resourced healthcare systems, the nurse- and AHP-led AI screening model holds transformative potential for global health. In low- and middle-income countries and remote or rural communities, traditional AF screening is often structurally impossible due to a shortage of cardiologists, prohibitive costs of standard 12-lead ECG machines, and lack of healthcare infrastructure. In these low-resource settings, the redistribution of healthcare tasks from highly qualified physicians to nurses and AHPs is a proven and necessary public health strategy (43). Equipping these frontline community workers with low-cost, AI-enabled tools, such as smartphone-based PPG applications or portable single-lead ECGs, can effectively democratizes access to advanced cardiac diagnostics. By leveraging existing community health networks, this decentralized model bypasses traditional infrastructural bottlenecks, facilitating early detection and anticoagulant initiation for the underserved populations. Consequently, widespread adoption of this model is not merely a workflow enhancement, but a critical mechanism for reducing global cardiovascular healthcare disparities (50, 51). Further research will be needed to validate AI-enabled devices and their clinical and economic benefits in real-world settings, including their impacts on long-term outcomes.

## 4 Conclusion

Nurse- and AHP-led AF screening in community and primary care settings is an effective strategy for improving the early diagnosis and management of AF. The use of AI-enabled devices to enhance the screening of undiagnosed AF holds substantial promise. Ultimately, the convergence of advanced AI diagnostics with the accessibility of nurse- and AHP-led care represents a vital paradigm shift in cardiovascular disease prevention. This model empowers nurses and AHPs to elevate their practice from operational screening tasks to comprehensive clinical leadership, care coordination, and proactive patient engagement.

To fully realise this paradigm shift, decisive action is required across three key domains:

- Implications for Practice: Healthcare facilities must move beyond ad-hoc screening and formally integrate AI-enabled AF detection into routine, nurse-led preventative care and chronic disease workflows, accompanied by protected time for comprehensive staff training.
- Implications for Policy: Policymakers and professional governing bodies must establish clear regulatory frameworks that support task-shifting, delineate liability protections for AI-assisted clinical decisions, and secure sustained funding and reimbursement models for community-based screening initiatives.
- Implications for Future Research: Future studies must pivot from controlled diagnostic accuracy trials to real-world implementation science. Priority should be given to longitudinal research that evaluates the long-term outcomes of stroke reduction, the clinical impact of mitigating algorithmic bias, and the economic viability of these decentralised models in diverse and low-resource settings.

AI-enabled AF screening devices have demonstrated significant clinical and economic benefits, including improved diagnostic accuracy, reduced healthcare utilisation, and cost-effectiveness. However, challenges such as data heterogeneity and implementation issues must be addressed to realise the full potential of these tools. Addressing these challenges will require a multifaceted approach that includes improving model transparency, enhancing data quality, providing education and training, upholding ethical standards, and ensuring adequate organisational support to fully transition to this new standard of care.
